# Executive functions and sustained attention: Comparison between age groups of 19-39 and 40-59 years old

**DOI:** 10.1590/S1980-57642012DN06010005

**Published:** 2012

**Authors:** Camila Rosa de Oliveira, Ana Cristina Pedron, Léia Gonçalves Gurgel, Caroline Tozzi Reppold, Rochele Paz Fonseca

**Affiliations:** 1 Psychologist, Master's Student at Pontifícia Universidade Católica do Rio Grande do Sul (PUCRS), Porto Alegre RS, Brazil. Supported by the Conselho Nacional de Desenvolvimento Científico e Tecnológico (CNPq). Clinical and Experimental Neuropsychology Research Group (GNCE).; 2 Psychologist, Master's Student at Universidade Federal de Ciências da Saúde de Porto Alegre (UFCSPA), Porto Alegre RS, Brazil. Supported by the Programa de Reestruturação e Expansão das Universidades Federais (REUNI); 3 Speech Therapist, Master's Student at UFCSPA. Supported by REUNI; 4 Psychologist, PhD, Associate Professor, Department of Psychology, UFCSPA. Supported by productivity grant 2F, CNPq.; 5 Speech Therapist, Psychologist, Adjunct Professor at the Psychology Department, Post-Graduate Program in Psychology, Human Cognition, GNCE, PUCRS. Supported by productivity grant 2, CNPq.

**Keywords:** age groups, executive function, inhibition, attention

## Abstract

Few studies involving the cognition of middle-aged adults are available in the international literature, particularly investigating the process of cognitive aging, executive components and attention.

**Objectives:**

The aim of this study was to investigate whether there are differences in performance on neuropsychological tasks of executive functions and sustained attention between two age groups.

**Methods:**

The sample consisted of 87 adults aged from 19 to 59 years old, divided into two groups according to the age variable (younger adults and middle-aged adults). All participants were Brazilian and had no sensory, psychiatric or neurological disorders; subjects also had no history of alcohol abuse, and no self-reported use of illicit drugs or antipsychotics. The neuropsychological instruments administered were the Hayling Test, Trail Making Test, Bells Test and verbal fluency tasks.

**Results:**

Groups showed no significant differences in relation to sociodemographic variables, educational level or frequency of reading and writing habits. The younger adult group performed better than the middle-aged group on tasks that involved mainly processing speed, cognitive flexibility and lexical search.

**Conclusions:**

These findings serve as a valuable reference for cognitive processing in middle-aged adults, since a large number of comparative studies focus only on the younger and later phases of adulthood. Additional studies are needed to investigate possible interaction between different factors such as age and education.

## Abstract (pt)

Na literatura internacional são escassos os estudos envolvendo a cognição de adultos de meia idade, principalmente quando o objetivo é investigar o processo de envelhecimento cognitivo, de componentes executivos e de atenção.

**Objetivos:**

Este estudo teve como objetivo verificar se existem diferenças no desempenho em tarefas neuropsicológicas que examinam componentes das funções executivas e da atenção sustentada entre dois grupos etários.

**Métodos:**

A amostra foi constituída de 87 adultos com idades entre 19-59 anos, divididos em dois grupos de acordo com a variável idade (adultos jovens e adultos de idade intermediária). Os participantes eram originalmente brasileiros e não apresentavam desordem sensorial (não corrigida), psiquiátrica ou neurológica, além de história de abuso de álcool, drogas ilícitas e antipsicóticos autorrelatadas. Os instrumentos utilizados foram o Teste Hayling, Trail Making Test, Teste de Cancelamento dos Sinos e tarefas de fluência verbal.

**Resultados:**

Os grupos não apresentaram diferenças significativas em relação às variáveis sociodemográficas, além de elevado nível de escolaridade e alta frequência de hábitos de leitura e escrita. O grupo de adultos jovens apresentou melhor desempenho do que o grupo de idade intermediária em tarefas que envolviam, principalmente, velocidade de processamento, flexibilidade cognitiva e busca lexical.

**Conclusões:**

Estes resultados fornecem referências para o processamento cognitivo também em adultos de idade intermediária, visto que um grande número de estudos comparativos foca apenas nas fases inicial e mais avançada da vida adulta. Estudos adicionais são necessários para investigar a possível interação entre fatores como idade e educação.

## INTRODUCTION

Neuropsychological assessment seeks to understand the complex relationship between brain organization, behaviour and cognition, being initially focused on researching cognitive deficits secondary to neurological afflictions such as brain damage. However, currently there is a growing body of research also directed toward the field of developmental neuropsychology, furthering understanding on the maturation, maintenance and decline of several cognitive functions (e.g. memory, attention, language) at different stages of the life cycle.

Although some theoretical models of cognitive processes have been well-developed and accepted in scientific circles, they are often based on extreme stages of life such as for children in the study of language acquisition^1-4^ and in aging after 60 or 65 years old, as in the investigation of memory decline.^5^ More specifically, the literature on cognitive aging, features studies comparing groups of older adults with younger adults.^6,7^ However, there are only a few studies available that have attempted to investigate the cognitive functioning of groups of adolescents and adults of intermediate age (40 to 59 years of age).^8-10^

Furthermore, executive function constitutes one of the cognitive components to which few studies on its relationship with biological and sociodemographic factors, such as age and education, have been dedicated.^6,11^ The concept of executive functions describes a group of cognitive abilities that control and regulate other process considered basic (such as attention, memory and motor skills). Executive functions are considered a comprehensive and complex construct.^12^ They are responsible for the ability to respond in an adaptive manner to new situations, involving skills such as inhibition, planning, and self-monitoring, among others.^13-15^ With regards to aging, Hamdan and Bueno^5^ observed the presence of alterations or deficits in verbal episodic memory and executive control, which were mainly associated with a decrease in information processing, attentional skills, inhibition and cognitive flexibility. Another study by Souza et al.^16^ evaluated 61 adults aged 19 to 70 years with at least seven years of schooling and investigated the hypothesis that executive behavior results from the integration of neuropsychologically simpler modules such as flexibility and planning ability. In this study, performance results demonstrated that executive neuropsychological functioning tended to decline with age and be facilitated by education. According to this evidence, Parente, Scherer, Zimmermann and Fonseca^17^ affirmed, in normative or comparative analysis among groups of normal individuals, that schooling was more relevant in most cases followed by, or on a par with, age factor. Also, not only did literacy influence cognition, but also the continuity of education combined with a series of cultural and social variables.

Yassuda et al.^18^ observed that the variables of age and education can significantly affect cognitive performance. According to these authors, cognitive changes were documentable from the fourth decade of life. Nevertheless, cognitive domains such as semantic memory and general knowledge about the world remain crystallized until a later age.

The investigation by Davis et al.^6^ compared the performance of verbal memory and the retention of individuals divided by age into four groups and concluded that performance was similar across all ages, except for the youngest group (30-45 years) and that the level of acquisition was lower in the two older age groups. LeBlanc et al.^19^ compared the physical and cognitive performance of individuals with mild, moderate and severe brain injury divided by age into three groups. These authors observed that older patients generally had worse outcomes in terms of global recovery in social participation, vocational and physical profiles compared to younger subjects. Therefore, the need for further studies in healthy adults compared to middle-aged subjects with reference data, plus more accurate clinical diagnosis, is evident. Among the few cross-sectional studies that have compared younger adults and middle-aged adults, the majority drew on normative data for components of attention and executive function.

However, in Brazil there seems to be insufficient research on the neuropsychology of adult development despite some concerted effort. Even in the international literature, studies on middle-aged adults' cognition are scarce, particularly when the aim is to investigate the cognitive aging process of executive and attentional components. The results of neuropsychological evaluations in this age range have been very heterogeneous, and therefore a challenge to be better understood by cognitive and developmental neuropsychology.^7,20^ This aim of this study was to verify whether there are differences in performance on neuropsychological tasks assessing components of executive functions and sustained attention between two age groups, namely, younger adults and middle-aged adults. The main hypothesis was that differences would be found in performance involving the executive components of initiation and inhibition, due to the known frontal hypothesis of a more probable age effect on frontal regions and connections, potentially leading to worse performance when inhibitory control is required.^21^ Moreover, these executive components are closely related to cognitive flexibility, generally affected by the age factor.^9^

## METHODS

**Participants**. The sample consisted of 87 adults aged from 19 to 59 years old, divided into two groups according to the age variable (younger adults, between 19 and 39 years, and middle-aged adults, between 40 and 59 years). Participants were from Brazil and had no sensory (auditory and/or uncorrected visual deficits), absence of psychiatric or neurological disorders, and no current or previous history of alcohol abuse or self-reported use of illicit drugs, benzodiazepines or antipsychotics.

In addition, subjects who had symptoms suggestive of depression as assessed by the Beck Depression Inventory - BDI (Brazilian version^22^), cognitive deficits as assessed by the Mini Mental State Examination - MMSE (Brazilian version^23^), and/or scores indicative of psychiatric disorders, investigated by the Self Report Questionnaire - SRQ (Brazilian version^24^), were not included in this study.

**Procedures and instruments**. This study was approved by the Ethics in Research Committee of the UFCSPA under number 913/09 and followed the ethical standards required by the resolutions on human research. The evaluation took place in an appropriate environment, and the sample was selected for convenience from university and business settings. The participants answered a battery comprising a sociodemographic questionnaire and neuropsychological tests in one session lasting approximately one and a half hours. The instruments were used to investigate components of executive functions and focused attention. The instruments were administered (in the order listed) as follows:

*Hayling Test* (Brazilian version^25^)- This assesses verbal initiation, planning and inhibition of automatic responses, besides processing speed.^26,27^ It consists of two parts: first, the participant is instructed to accurately complete, as fast as possible, incomplete sentences, while in the second part, he/she must also complete sentences, but providing answers that do not have any semantic relationship with the phrase. Dependent variables were reaction time (in seconds) for the provision of responses in terms of test time on each part (Part A and B) and number of errors. The difference between time on Part B and A is also calculated in order to verify inhibition ability.

*Trail Making Test* - TMT^28,29^- This measures processing speed, cognitive flexibility, visual search and motor performance.^30^ It is also composed of two parts: in the first part, the participant is instructed to join a sequence of numbers in ascending order; and in the second part, he/she must join the dots between numbers and letters, in alphabetical and numerical order, respectively. For example, the participant starts at number 1 and must draw a line to the letter A, then continue from A to the number 2 and onto the letter B and so on. Dependent variables were reaction time, in seconds, to complete each part of the test (Part A and B) and number of correct answers and errors made in each part. The difference between the times taken on Part B and A is also calculated in order to assess divided attention.

*Verbal Fluency Tasks* (Brazilian version^31^)- On these tasks, participants have to evoke as many words as possible that are not proper nouns or numbers for three types of verbal fluency (free, lasting two and a half minutes; with an phonemic/orthographic criterion - only words starting with the letter P, lasting two minutes; and with a semantic criterion - only words for objects that are worn or are clothing, lasting two minutes). The dependent variable is the number of words recalled correctly on each of the modalities of verbal fluency.

*Bells Test* (Brazilian version^32^)- This task provides measures of sustained and selective attention as well as visual perception and processing speed. In this test, participants must find and cancel the target figures (bells) which are distributed pseudo-randomly among other visual distractors. The dependent variables are search time in seconds needed to find the target stimuli (bells), number of omissions and distractors marked.

**Data analysis**. All data were parametric according to the one-sample Kolmogorov-Smirnov Test (p>0.05). Thus, mean accuracy and time scores were compared between groups by means of Student's *t* test for independent samples (p≤0.05). The statistical package used was SPSS 15.0.

## RESULTS

The groups of younger adult and middle-aged adults showed no significant differences in relation to sociodemographic variables (Table 1), indicating that they were matched. Participants had a high educational level as well as a high frequency of reading and writing habits.

**Table 1:** Sociodemographic characteristics by group.

Variables	Younger adults			Middle-aged adults		df	t
	**M**	**SD**		**M**	**SD**		
Age (years)	26.17*	6.10		48.59*	4.93	85	-18.56
Education (years)	12.17	2.60		12.23	3.00	85	-0.11
Frequency of reading and writing habits	16.52	5.25		17.13	4.75	84	-0.56
MMSE	29.52	0.97		28.97	1.55	85	1.92
BDI	3.62	4.81		4.69	5.35	85	-0.98
SRQ	2.98	3.26		2.39	2.53	82	0.90
N	48	39					

df: Degrees of freedom;

* p≤0.001.

## DISCUSSION

This aim of this article was to investigate differences in performance between two age groups (younger adults and middle-aged adults) on tasks assessing sustained attention and components of executive functions. The group of younger adults studied showed better performance on 50% of the variables measured, especially for those requiring processing speed, cognitive flexibility and the ability to produce words from a semantic criterion.

In relation to decline in cognitive abilities during the life cycle, Moraes et al.^33^ noted that working memory, processing speed and visuospatial skills suffer greatest change with increasing age. Similar results were found by Fonseca et al.^7^ and Davis et al.^6^ in the performance of younger adults on the TMT and Hayling test compared to elderly subjects. According to a review by Sánchez-Cubillo et al.,^34^ several studies using TMT to investigate measures of visual search, perceptual and motor speed, processing speed, working memory and general intelligence have been carried out. Part B of the test is responsible for assessing the executive components of cognitive flexibility, inhibitory control, and working memory. However, according to Periáñez et al.,^30^ the TMT was not sensitive for distinguishing populations of younger adults and middle-aged adults. Despite this criticism, the instrument proved sensitive to differences between the younger groups in this study.

Regarding the results obtained by comparing the Hayling Test, the formula (time B - A) is used to investigate the rate of suppression of automatic responses and has shown greater sensitivity for the discrimination of age groups. Within the broad range of studies on pathologies related to aging, the Hayling Test has been administered (largely in populations with dementia in order to detect inhibitory deficits^35-37^), demonstrating its clinical validity.

Based on studies comparing the performance of young adults and elderly adults on neuropsychological tests, Banhato and Nascimento^38^ found better performance among young adults (20 to 34 years) on executive tasks, detecting slight loss in working memory and moderate loss in attentional skills, processing speed and visuospatial organization. The results of this study, although it did not consider the comparison between extreme age groups, suggested a possible onset of decline in cognitive functions linked to inhibitory components and processing speed.

Accuracy proved to be the least sensitive variable for differentiating the groups. On the Bells Test this result cannot be explained by the complexity of the task, since participants were highly educated. However, in relation to the verbal fluency component, a smaller number of words produced by the group of middle-aged adults suggests an early decline in the initiation process and lexical search. For the different forms of verbal fluency (free, orthographic and semantic), differences in the level of complexity required in each of these were present, and semantic verbal fluency appears to have the highest sensitivity for distinguishing age groups.

Studies such as that by Rodrigues et al.^39^ and Mathuranath et al.^40^ have concluded that semantic verbal fluency requires greater activation of the temporal lobe regions and depends on the access and integrity of semantic memory, a component of long-term memory that contains the permanent representation of our knowledge about objects, facts and concepts, as well as words and their meanings. On the other hand, recent findings suggest that more frontal connections are activated during the execution of semantic verbal fluency tasks than was initially thought, suggesting a greater role of components of executive functions in this cognitive task.^41-43^ According to Tombaugh et al.^44^ performance on semantic verbal fluency tasks was more strongly associated with age. However, Peña-Casanova et al.,^45^ when comparing different age groups from 50 years old, failed to confirm this association. One hypothesis is that, in general, the category used in verbal fluency tasks is animals, possibly more familiar and extensive than clothes. Since clothing is possibly a harder category, this modality chosen in the present study may have shown better discrimination by demanding more executive search of lexical components in the interaction with language and mnemonic processing.

In general, the hypothesis of differences in the ability of inhibition and initiation being more strongly related to processing speed than to accuracy was confirmed, as middle-aged adults performed worse than young adults particularly for the variable of execution time on both the Hayling Test and Trail Making Test. These findings serve as a valuable reference for cognitive processing in middle-aged adults, where a large number of comparative studies have focused only on the initial and final stages of adulthood.

However, the relatively small sample and wide range of age groups are limitations to be considered in this study. In addition, further studies are needed to investigate the possible interaction between the factors age and education. The results of this comparative study, although preliminary, show evidence pointing to the importance of conducting evaluations in the intermediate age population. Through such evaluations, deficits and dissociations regarding different cognitive processes, particularly attention, memory, executive functions and language, can be diagnosed earlier and more accurately.
